# Is this the ‘new normal’? A mixed method investigation of young person, parent and clinician experience of online eating disorder treatment during the COVID-19 pandemic

**DOI:** 10.1186/s40337-021-00429-1

**Published:** 2021-06-30

**Authors:** Catherine Stewart, Anna Konstantellou, Fatema Kassamali, Natalie McLaughlin, Darren Cutinha, Rachel Bryant-Waugh, Mima Simic, Ivan Eisler, Julian Baudinet

**Affiliations:** 1 Maudsley Centre for Child and Adolescent Eating Disorders, Maudsley Hospital, De Crespigny Park, Denmark Hill, London, SE8 5AZ UK; 2 Institute of Psychiatry, Psychology & Neuroscience (IoPPN), King’s College London, 16 De Crespigny Park, Denmark Hill, London, SE5 8AF UK

**Keywords:** Child, Adolescent, Online therapy, Telehealth, Anorexia nervosa, Bulimia nervosa

## Abstract

**Introduction:**

Prior to the COVID-19 pandemic, research in virtual care for young people with eating disorders was preliminary and implementation rare. This study explored the experience of young people, parents and clinicians when therapy was transitioned to virtual provision as a result of the UK lockdown in March 2020.

**Methods:**

A mixed-method approach was used in this study. Online questionnaires that included a mixture of rating (Likert scale) and free-text response questions were completed by 53 young people with any eating disorder, 75 parents and 23 clinicians. Questions focused on the experience of online treatment as well as the impact on engagement, perceived treatment efficacy and preferences around treatment mode in the future. Likert scale questions were analysed using a summary approach. Free-text responses were analysed qualitatively using reflexive thematic analysis.

**Results:**

Responses to rating scale questions indicate satisfaction with treatment, good engagement and ability to manage technology. Young people who had transitioned care, rather than started care virtually in lockdown, rated therapy as less effective. However, individual accounts of experience were more varied. Reflexive thematic analysis of free-text responses identified key themes of 1) Making it work, 2) Home as a therapeutic space, and 3) Disrupted connection and 4) Into the future.

**Conclusions:**

These results have implications for ongoing care during the pandemic and for future implementation of virtual care in the treatment of young people with eating disorders. Particular issues arising are the trade-off between accessibility and therapeutic engagement and depth and need for consideration of equal access to treatment in socially unequal societies.

**Supplementary Information:**

The online version contains supplementary material available at 10.1186/s40337-021-00429-1.

## Plain English summary

Due to the COVID-19 pandemic specialist eating disorder treatment needed to quickly adapt to new technologies. All but urgent and crisis appointments needed to be delivered online. This study looked at what that was like for the young people, their parents and clinicians engaged in treatment in a large specialist eating disorder service in London, UK. Fifty-three young people with an eating disorder, 75 parents and 23 clinicians completed anonymous online surveys about their experience. Results showed that generally people were satisfied with treatment, the therapeutic relationship was maintained and most managed technological issues well. Four main themes were identified from responses to open ended free-text response questions. All three groups wrote about 1) making it work, 2) home as a therapeutic space, and 3) disrupted connection. The fourth theme, 4) into the future, came from parent and clinician responses only. These results have implications for ongoing care during the pandemic and for future implementation of virtual care in the treatment of young people with eating disorders. Particular issues arising are the trade-off between accessibility and the potential impact on the therapeutic relationship. There is also a need to make sure there is equal access to treatment in socially unequal societies.

**Supplementary Information:**

The online version contains supplementary material available at 10.1186/s40337-021-00429-1.

## Introduction

As a result of the COVID-19 pandemic, mental health care, including care for people with eating disorders, has had to rapidly transition to virtual delivery [1–3]. Reports indicate the exacerbation of symptoms for existing patients [4–6], and an increase in referrals to eating disorder services across age groups [7]. For children and adolescents specifically, the disruption to routines, social isolation, perceived lack of control and physical health anxieties have been linked to increased eating disordered cognitions and adverse mental health outcomes [8]. Together with the continuing need for virtual delivery a year after the start of the pandemic, it is vital that we understand the impact that virtual working has on young people, families and clinicians. This will ensure ongoing provision is responsive to people’s needs and informs post pandemic developments in virtual interventions for eating disorders.

Research conducted prior to the pandemic suggests that online psychological interventions can achieve satisfactory feasibility, acceptability and treatment outcomes for young people with restrictive eating disorders [9], albeit with very small sample sizes and in the context of expanding opt-in care to rural or remote areas in North America. This is clearly markedly different to an enforced pivot to virtual care during a pandemic in the UK, where access to Community Eating Disorders Services for Children and Young People (CEDS-CYP) has been a focus of nationwide development in recent years [10].

Since the start of the pandemic, emphasis has been placed on telehealth interventions to treat young people [11]. In response, early publications included expert clinical opinion, guidance documents and literature reviews [12, 13]. Preliminary recommendations endorsed the use of telehealth and encouraged increased communication between teams, clear expectation setting for service users, competence with the necessary technology and familiarity with national guidance to ensure the feasibility of telehealth [14, 15].

Early evidence indicates that several services were able to adapt child and adolescent eating disorder treatment to virtual delivery. Publications described the adaptations made to facilitate online assessment, treatment, and weight restoration alongside reports of consequent operational improvements and pressures [1, 11, 16].

Overall, the literature suggests that patients were willing to undertake virtual therapy and recounted largely positive experiences with their eating disorder treatment [1, 17, 18]. Some studies further suggested unforeseen benefits of the adapted method of working, such as a greater level of familial involvement in treatment [18, 19] and advocated for its use beyond the pandemic to facilitate greater access for individuals in rural areas [20]. In one UK based CEDS-CYP, treatment satisfaction was higher for young people (*n* = 12) and parents/carers (*n* = 19) compared to clinicians (*n* = 12), regarding online working during the pandemic [1]. Several clinicians expressed concerns about the therapeutic relationship, difficulties adapting family-based treatment, novel pressures and potential therapist burnout. Therapist reticence in using virtual delivery, associated with both logistical barriers and concerns about appropriateness, has been highlighted as a barrier to telehealth implementation [21].

Despite early reports of engagement and potential benefits of virtual working, concerns remain regarding the privacy and accessibility of telehealth [22, 23] as well as the potentially detrimental impact on recovery of social isolation and economic strain in the context of COVID-19 [24, 25]. Disparities in patient engagement with online interventions have previously been reported in relation to socioeconomic status, race and literacy [26]. However, some have argued that the pandemic should be taken as a ‘black swan’ – rare, but predictable retrospectively – and push us to a place of change which was inevitable with hindsight [27].

The research presented here sought to address gaps in the existing literature around young person, family and clinician experience of telehealth following the transition of an entire CEDS-CYP to virtual delivery in response to COVID-19 lockdown in the UK. The mixed method study of a large group of families and clinicians in a well-established service enables comparison of the experiences of these different stakeholders. This allows for reflection on maintaining quality as well as continuity of care as the pandemic continues. It further provides opportunity for transferable learning beyond the immediate crisis and into a period of potential increase in the use of telehealth in eating disorder care internationally.

## Methods

### Sample

All clinicians, young people and parents who were working in or receiving treatment at the Maudsley Centre for Child and Adolescent Eating Disorders (MCCAED) outpatient service during the data collection period were invited to participate. All young people had a diagnosis of an eating disorder (typical or atypical Anorexia Nervosa, Bulimia Nervosa, Avoidant Restrictive Food Intake Disorder or Other Specified Feeding and Eating Disorder). No demographic data were collected, and responses were not linked to clinical records in order to ensure anonymity. Young people were all less than 18 years of age at the time of data collection. All clinicians were qualified family therapists, psychiatrists, nurse therapists or clinical psychologists with training and experience in treating eating disorders. The young person and parent experience of moving day programme treatment to virtual working in the same service is presented elsewhere [18].

### Treatment setting

MCCAED provides specialist treatments for children and adolescents with eating disorders for an area of South East London with a population of approximately 2 million people. The outpatient service provides specialist family and psychological therapies, psychiatric management, dietetics and physical health reviews. The primary treatment model is eating disorder focused family therapy, although other evidence-based treatments are also offered. Treatment for children and adolescents with ARFID follows current international consensus guidance recommending multi-disciplinary, multi-modal input. Due to the COVID-19 pandemic, all but essential face-to-face contact was ceased. Essential contact included assessment and management of high physical or mental health risk. All outpatient treatment was delivered via video/phone calls.

### Research governance

This study was approved as a clinical service evaluation/audit by the South London and Maudsley NHS Foundation Trust CAMHS audit committee.

### Procedure

Online surveys were used to collect data from young people, parents and clinicians via the Qualtrics XM online platform [28]. The use of surveys is an underutilised but valid qualitative research tool [29]. This format allowed for data collection at a time when face-to-face contact was prohibited and ensured a high degree of anonymity for participants. This allowed for participation of all clinical staff within the service, giving voice to a range of perspectives that may have been lost in online focus groups or online interviews, where existing relationships and power structures may have influenced people’s responses. Data were collected between 18th May and 25th July 2020. This was within the first few months of the first UK lockdown which took effect on 26th March 2020. Each patient was classified as either an established patient (EP; five or more face-to-face sessions prior to lockdown) or a new patient (NP; fewer than five face-to-face sessions prior to lockdown or assessed in lockdown).

### Surveys

#### Clinicians

Likert scale rating questions were used to assess therapists’ sense of efficacy and comfort working online (1 not at all – 10 definitely/very), and how it compared to face-to-face work (1 less, 5 the same, 10 more). This was followed by six open-ended questions, which explored the experiences of working online (see Supplementary Material, Table 1). Therapists reviewed each case on their caseload and rated engagement with the family and young person prior to and since moving to online therapy.

#### Young people and family

A similar format was used for young people and parents. Likert scales were used to assess overall treatment experience, how understood people felt, the degree to which important issues were able to be addressed, the impact of technology, and perceived efficacy of online therapy (1 poor/not at all – 7 definitely). Participants were also asked about their preferences for treatment once restrictions eased (online, in-person, or dependent on needs). Four open-ended questions were also included to explore perceived difficulties and advantages of online therapy and suggestions for improvements.

Those in the EP group were asked additional questions focused specifically on the transition from face-to-face to online treatment. The impact of this transition on the therapeutic relationship, quality of therapy, perceived benefit from online therapy (Likert scales 1 not at all– 7 definitely) and impact on recovery (1 detrimental – 7 positive impact) were assessed. There were two further open-ended questions asking about losses and gains from the move to online therapy.

### Analysis plan

#### Quantitative analysis

A series of non-parametric tests were conducted on the Likert scale rating questions. Changes in clinician rated engagement for EP were analysed via Wilcoxon Signed Ranks tests. Differences between the experiences of EP and NP were assessed via Mann Whitney U tests.

#### Qualitative analysis

Data were analysed using reflexive thematic analysis within a critical realist framework, which views meaning and experience as subjective and influenced by social and cultural context [30, 31]. Comments were initially coded, then topics defined. Themes were then developed through reflexive engagement with the data [30], with the intentional involvement of two authors who were both working as therapists in the service at this time (CS and JB). This facilitated discussion and reflection around the impact of the social and cultural context of the team and work, as well as the broader impact of the pandemic in South East London. Details of the process can be seen in Supplementary material Table 2.

## Results

### Sample

Electronic links to surveys were sent via email to 163 young people, 161 parents and 35 clinicians. Of these, 53 (33%) young people (NP *n* = 21, EP *n* = 32), 75 (47%) parents (NP *n* = 40, EP *n* = 35) and 23 (66%) clinicians responded. Clinicians provided ratings of engagement for their entire caseload, not just for the families participating in this study. This included ratings for 212 young people and 193 families.

### Quantitative

Descriptive and inferential statistics for all participants are included in Table 1.

**Table 1:** Clinician, young person and parent ratings of engagement and treatment experience

***Clinicians***					
	*Pre-lockdown*		*Post lockdown*		
	*Mdn. (IQR)*		*Mdn. (IQR)*		*Test statistics*
Engagement with YP (*N* = 170)	8 (2.75)		8 (3)		*z* = −1.36, *p* = .17
Engagement with parents (*N* = 156)	8 (3)		8 (2)		*z* = −0.51, *p* = .61
	*EP*		*NP*		
	*N*	*Mdn. (IQR)*	*N*	*Mdn. (IQR)*	
Engagement with YP	128	8 (3)	63	7 (3)	*U* = 3794.00, *p* = 0.50
Engagement with parents	119	7 (3)	57	8 (2)	*U* = 3121.50, *p* = 0.39
***Young people***					
	*EP (N = 32)*		*NP (N = 21)*		
	*Mdn.* (IQR)		*Mdn.* (IQR)		
Overall experience	5.0 (2)		6.0 (3)		*U* = 258.00, *p* = 0.15
Feel understood	5.0 (3)		6.0 (1)		*U* = 229.00, *p* = 0.08
Address important issues	5.0 (2)		6.0 (2)		*U* = 160.50, *p* = 0.002**
Impact of Technology	2.5 (2)		4.0 (3)		*U* = 242.00, *p* = 0.08
Benefited from online	5.0 (3)		7.0 (3)		*U* = 126.00, *p* = 0.02*
***Parents***					
	*EP (N = 35)*		*NP (N = 35)*		
	*Mdn.* (IQR)		*Mdn.* (IQR)		
Overall experience	6.0 (2)		6.0 (3)		*U* = 651.00, *p* = 0.64
Feel understood	7.0 (1)		6.0 (1)		*U* = 656.00, *p* = 0.57
Address important issues	6.0 (2)		6.0 (2)		*U* = 758.50, *p* = 0.12
Impact of Technology	4.0 (4)		4.0 (2)		*U* = 643.00, *p* = 0.88
Benefited from online	6.5 (2)		7.0 (2)		*U* = 368.00, *p* = 0.50

*Abbreviations: EP* established patients, *Mdn.* median, *IQR* interquartile range, *NP* new patients, *YP* young person

* = p < .05; ** = p < .01

#### Clinician perspective

##### Clinicians’ ratings of engagement

No significant differences were found in clinician ratings of engagement with young people or parents in the EP group prior to and 2 months into lockdown, suggesting that clinicians felt that levels of engagement were not changed by the transition to online therapy.

No significant differences were found in clinician rating of engagement post lockdown between EP and NP young people and parents, suggesting that clinicians felt that young people and parents could be as effectively engaged online as with face-to-face treatment.

##### Clinician’s experience of online therapy

While overall there was a tendency for clinicians to consider themselves to be relatively efficacious (*Mdn. = 7, IQR* = 2) and comfortable working online (*Mdn. = 7, IQR* = 2), there was a wider range of scores when asked to compare this to usual face-to-face practice (Efficacy *Mdn. = 4, IQR* = 3; Comfort *Mdn. = 4, IQR* = 3). There was also a more variable experience of the impact of technology on therapy (*Mdn. = 4, IQR* = 4).

##### Young people and parents’ perspective

Young people and parent’s scores indicated that their overall experience of online therapy was positive and that they felt understood, with a low level of impact of technology on their treatment experience. Analysis of young people’s ratings did not reveal any significant difference between the EP and NP groups, with the exceptions of perceived treatment benefit and how much they felt important issues could be addressed online. The NP group’s ratings of these were significantly higher than the EP group’s. Analysis of parents’ ratings did not reveal any significant difference between EP and NP groups. See Table 1 for further details.

Both parents and young people in the EP groups indicated little impact on the therapeutic relationship (Young people *Mdn. =* 1, IQR = 2; Parents *Mdn. =* 1, IQR = 1). Young people’s rating of the quality of therapy was more towards the mid-point indicating that some were identifying a change in quality (*Mdn. =* 3, IQR = 3). This was not reported by parents (*Mdn. =* 1, IQR = 3). Both young people and parent’s rating of the impact on recovery was also at the mid-point, indicating that, for some, the change to online therapy was perceived as impacting on recovery (Young people *Mdn. = 4*, IQR = 2; Parents *Mdn. = 4*, IQR = 5).

There were significant differences between young people and parents in preferences for treatment format once restrictions are eased (*χ*^2^ (2, 120) = 6.93, *p* = .03). Post hoc analyses revealed that this was driven by parents being more likely to opt for online appointments over face-to-face than young people (*χ*^2^ (1, 57) = 6.12, *p* = .01). See Table 2.

**Table 2:** Young people and parents’ preferences for continued online once COVID-19 related restrictions ease

	Young people (*N* = 52)		Parents (*N* = 68)	
–	*N*	*%*	*N*	*%*
Yes, I would like to continue to meet with my/my child’s therapist online	3	5.8%	12	17.6%
No – I would like to have appointments with my/my child’s therapist face to face	24	46.2%	18	26.5%
I don’t mind – I would be happy with either depending on what my/my child’s needs are	25	48.1%	38	55.9%

### Qualitative

**Fig. 1:**
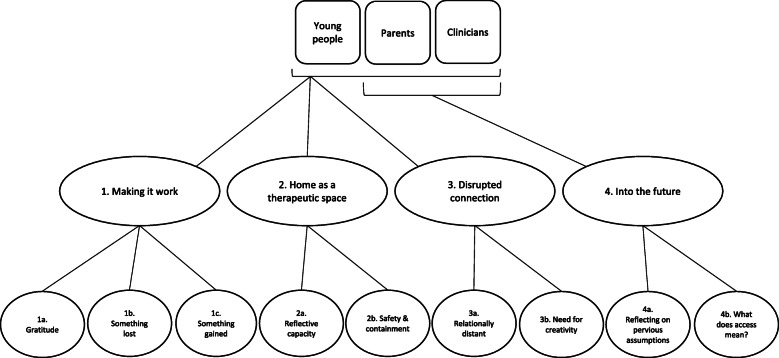
Thematic map of qualitative analysis

Analysis of free text responses revealed three main themes and six subthemes that applied to all young people, parents and clinicians (see Fig. 1). These were 1) Making it work, 2) Home as a therapeutic space, and 3) Disrupted connection. An additional theme, 4) Into the future, and two subthemes, were identified for the parents and clinicians only. Each are described further below with relevant illustrative quotations.

#### Making it work

##### Gratitude

Generally, there was an overwhelming sense of gratitude from all. People felt fortunate to be able to provide and access a service online and appreciated the continuity of care. This was echoed by newer families as well as those in more established therapeutic relationships.

*Young person: “I do not think that there is anything more [the team] can do. They are doing a great job and are supporting me well”*

*Parent: “It did not stop! We are very grateful we did not need to pause it and it feels like an extension of what we had”*

##### Something lost

Despite this feeling of gratitude, the transition to online working was difficult for many. The majority said treatment continued to be helpful and there was a sense that it was ‘better than nothing’ given the circumstances. However, it was clearly expressed that it was not the same, that face-to-face working was preferable, and at least ‘a few’ face-to-face sessions were wanted to build rapport. One clinician noted: *“Every patient I see has said they prefer face-to-face”.* Many parents also noted that their children felt less comfortable with online working than they did. Young people expressed “missing” their therapist and that the process felt impersonal.

*Young person: “It has made it harder to connect with [the therapist] and more awkward talking about actual problems.”*

*Parent: “Therapy is a very personal thing, doing it online is hard because it's hard to read body language, emotion etc. It's better than nothing but it's not as good as face-to-face by a long way”*

*Clinician: “I find the family sessions harder and sometimes feel like I am missing nuances and process issues”*

For clinicians, there was also a sense of loss around team working and the social elements of being in a multi-disciplinary team.

*Clinician: “I am finding the lack of spontaneous and informal colleague input very difficult, and it can feel very lonely, which impacts on my confidence and sense of efficacy”*

##### Something gained

Despite several aspects feeling disconnected, this was balanced by new discoveries and advantages. Some said that the relationship with their therapist remained stable and that they hadn’t noticed obvious losses associated with online therapy. Many commented on the increased access and convenience afforded by online working. Some parents noted that it allowed both parents, or more family members, to attend more regularly, which was perceived as helpful. Clinicians noticed professional growth in a relatively brief period, describing a period of challenge and struggle from which they were emerging with new confidence, skills and tools.

*Young person: “[being online] meant I could do it in my pjs [and] couldn't see my body, which reduced some anxiety about the situation”*

*Parent: “Online has worked in our favour … It couldn’t have come at a better time. It meant we were able to give it great focus without interruptions. [My child] made enormous rapid progress”*

*Clinician: “I have days when I feel I am losing confidence as a therapist. Other days I feel OK and am amazed by what can be achieved despite never having met a patient or their family”*

#### Home as a therapeutic space

All young people, parents and clinicians observed a unique impact of attending treatment from home. This affected both the practical and process aspects of treatment.

##### Reflective capacity

Most people noticed that increased accessibility reduced treatment burden, which promoted new thinking and comfort. However, many also noted it was difficult to differentiate the therapeutic space from home-life. For some this made it harder to reflect on and prioritise treatment, while for others the change of environment brought new reflections into therapeutic conversations.

*Young person: “Sometimes having a different setting acted as a 'fresh start' and [we] were able to look at situations from a different perspective”*

Parent: “The illness and the treatment are always in [the] same environment which can fade out light bulb moments because your perspective doesn’t change”

*Clinician: “seeing patients’ home environment and the families in their home settings provides significant information and facilitates formulation”*

##### Safety and containment

Being at home also impacted therapeutic safety and containment. There was a strong expression of concern about privacy and being overheard, which was particularly voiced by the young people.

In what was an anxiety provoking time for all, parents voiced fear that the therapist could not really know how the child was progressing and some requested increased contact between sessions, more feedback about separated session content and written summaries of family sessions.

Parents described a shift in responsibility for some aspects of their child’s recovery. This was very apparent through the need for parents to weigh their child at home, and therefore manage falsification and unreliable scales. Shift in the power dynamics between young person, family and therapist were described. For some parents this was uncomfortable as they experienced being perceived as too controlling by the child. For others, the shift to greater responsibility or perceived equality with the therapist was welcomed. Some young people also described an increased sense of responsibility for their recovery.

Many clinicians described elevated feelings of exhaustion, increased isolation from their colleagues, and heightened uncertainty when needing to make decision. Some therapists expressed a reduction in confidence and concerns about treatment efficacy. For many, privacy was a major source of concern, with some struggling to set new boundaries between work and home.

*Young person: “It doesn't feel as much of an enclosed, safe space as it does when you are allocated a 'therapy room' at the centre”*

*Parent: “It is very difficult for us to weigh our daughter weekly and have an accurate number. My daughter has been tricking me with her weight and this delays her recovery. “*

*Parent: “A private space to talk, [she] worries about family members hearing what she talks about. We live in a flat so that has been hard for her.”*

*Clinician: “I think the distance that online creates means that I have on a couple of occasions taken risks in what I have said when I wouldn’t have in person. This has left me feeling anxious and unable to gauge the impact of what I have said”*

*Clinician: “Therapists’ privacy can be an issue with exposure of their home environment; the background pictures for this can feel defensive and make patients uncomfortable”*

#### Disrupted connection

Almost all participants noted changes in the therapeutic relationship. This change was attributed to many different things, including lack of privacy at home, technology issues, being more distractible, and loss of some process aspects of therapy (e.g., body language, position in the ‘room’, use of silences/pauses). Others noted that treatment felt ‘less real’ and that it was harder to remember appointments and what was discussed in session.

##### Relationally distant

Most young people, parents and clinicians reported some kind of relational disconnect in online treatment. People reported being less honest, struggling to read body language, uncertainty about pauses and a tendency to revert back to problem solving instead of staying with emotions. This led to several young people and clinicians querying the efficacy of online working. The parents of young people who were newer in therapy described feeling less personally connected. These parents were more likely to express concern about the extent to which their children were able to engage through the virtual medium.

*Young person: “Slightly harder to form an as close relationship with therapist and [it is] easier to be fully open in person”*

*Parent: “Generally I think it is harder for all parties to make a direct connection - with communication sometimes becoming too direct and less empathic. It is harder for all concerned to miss more subtle body language.*

*Clinician: “I miss being in the room with people and being able to engage with them and them with me using our whole bodies and all the expression that goes with that. I feel distant from families and sometimes unable to reach them”*

Not everyone felt this way. Parents tended to be more comfortable within the online therapeutic relationship than young people or clinicians. A minority of young people also noted that their therapeutic relationship improved.

*Young person: “My relationship moved forward as it would have done in a face-to-face setting i.e., wasn't affected by the online treatment”*

##### Need for creativity

There was a sense from several people, particularly clinicians, that online working needed creativity and adaptation. Many remarked on certain elements of treatment now feeling “*clumsy*”, “*awkward*”, or “*strange*”, when elements of face-to-face working were directly replicated for online working. For example, several people noted that seeing people’s faces close-up disrupted the usual therapeutic process and made it feel more like a “*business meeting”* (clinician). Others found it difficult to replicate written or drawing tasks. It was felt that creativity was needed in both establishing and maintaining therapeutic relationships, as well as for the tasks of therapy itself. Clinicians and parents both highlighted the particular needs of younger, more active children and those with neuro-developmental difficulties.

*Clinician: “Creative techniques are more challenging to use (although not impossible) … sitting next to someone to draw a genogram or a lifeline is an intervention at both verbal and non-verbal communication levels which cannot be replicated online”*

#### Into the future (parents and clinicians only)

Parents and clinicians both referred to implications for the future.

##### Reflecting on previous assumptions

Many reflected on revisiting certain assumptions about online treatment and the recovery process. Several came with preconceived beliefs and found this way of working challenging and surprisingly rewarding. Clinicians raised concerns about virtual working without team contact in the office, for example around the integration and training of new team members in the future.

*Parent: “ultimately, responsibility lay with the carers of the person suffering. So although the illness is relentless, we know we have to get on and possibly having no ‘face-to-face’ support instils that message further.*

*Clinician: “ [I] think it challenged some beliefs about efficacy and the therapeutic relationship in a positive way”*

##### What does access mean?

The trade-off between increased access and more stilted connection left parents and clinicians wondering which parts of treatment are most important. Several noted that treatment felt more practically focused and questioned whether this would impact on efficacy. There was also some consideration of whether this format may suit different treatment tasks (e.g., refeeding) over others (e.g., identity exploration).

Accessibility to treatment was not universal. The negative impact on the process of therapy and increased stress around accessing appointments for families for whom the technology did not work well highlighted the poorer quality of therapeutic experience for some families.

*Young Person: “When I’m having a deep conversation of telling something important and then the screen glitches and my therapist hasn’t heard any of it”*

*Parent:” I think it’s worked excellently for the refeeding programme. But …there are issues [my child] needs to talk about that are the root cause of her illness and I can’t see her opening up in this situation”*

*Parent: There might need to be consideration given to families that have IT access issues and a pre-paid (by the council) broadband connection offered to low-income/ vulnerable families to benefit from online therapy.”*

*Clinician: “I feel like the treatment I give is less efficacious. It slips back into problem solving more often than dealing with and managing strong affect and process”*

## Discussion

This mixed method study explored the experience of online working in a UK specialist CEDS-CYP service early in the COVID-19 pandemic. A reflexive and critical realist approach was taken in analysis of the data, drawing on the authors’ experiences of being clinicians during the COVID-19 pandemic, the existing literature around online therapy for eating disorders and the potential for current online treatment practices to influence future CEDS-CYP service developments.

A synthesis of the experience of young people, parents and clinicians identified that most had an appreciation for the continuity of care afforded by the online platform. Most young people and parents also appreciated the increase in treatment accessibility, convenience and comfort. However, there were also feelings of disconnection and reduced containment. Generally, there was also a trend for responses to rating scale questions to reflect a more positive experience than free-text responses.

Young people were more likely to describe the experience as difficult and felt more disconnected than parents. The overall experience of online working was also rated lower by those who had had the experience of face-to-face treatment, reflecting a sense of loss through the transition. Parents and clinicians also experienced disconnection, alongside a recognition of some clear logistical gains and unexpected therapeutic benefits. The shift in roles for clinicians and parents was experienced as motivating but also as isolating and anxiety provoking.

Many clinicians found the move to working from home isolating and reported a decrease in confidence. This context is different to offering online therapy from a clinic, for which therapists may be trained and delivering by choice. Many described increased levels of uncertainty and feeling as though team members were less available for more informal consultation and discussion. Concerns were raised about management of medical and safety risk issues although there were clear protocols in place and daily planned contact made available with senior therapists and medical doctors. Therefore, concerns around risk management need to be interpreted within the context of reduced therapist well-being and limited face-to-face team contact.

Several clinicians experienced working from home, a previously private space, as intrusive. Some noticed this impacting upon their sleep and stress levels. This shift challenged deeply trained stances around personal disclosure and availability. This may be a particular consequence of pandemic related restrictions, but in the context of financial pressures on health services might require consideration when asking therapists to work from their homes in the future.

The discussion around access highlighted the intersection between therapeutic safety and socioeconomic factors. The risk was clearly described around poorer therapeutic experience for those with less access to technology and private space at home. Many people described using phones as their main device, struggling with weak internet connections and living in relatively small spaces which led to reduced engagement in therapy as a direct result of fearing being overheard.

The availability of online therapy was mirrored by a feeling of distance and being able to hide. Essentially, increased access did not always mean increased connection, and many had a strong preference for at least some face-to-face working. Some elements of comfort, such as not having to be seen or not leaving the house, could also facilitate avoidance and might become therapeutically detrimental. This is pertinent to the potential ongoing use of virtual treatment of adolescents with eating disorders. Given that increased therapeutic alliance is associated with improved outcomes in child and adolescent psychotherapy [32], and eating disorder focussed family therapy specifically [33], this will be important to consider when designing future online treatments.

Both technological and relational disconnections had an impact on the form that therapy took in this period, with most participants describing difficulty expressing or discussing emotions online. As a result, treatment felt more content focused than process driven. Several participants questioned whether this would impact on treatment efficacy.

### Strengths and limitations

The mixed methods design and inclusion of young people, parents and clinicians provides a richness to the data. This has helped to determine similarities and differences between experiences of different stakeholders. The use of a qualitative survey is a particular strength of this research as it allowed for a relatively large sample size, which included a range of voices and experiences. Views could be expressed anonymously without the constraints of power dynamics inherent in asking both service users and clinicians about their experience of their treating service or workplace.

The generalisability of these findings to future developments of virtual provision of mental health care must be evaluated with caution considering the unique context of the pandemic and the point during which data were collected. All participants were living in a time of heightened uncertainty, anxiety and stress, and reduced social interaction. Very little was known about COVID-19 at the time. Now that people are more accustomed to life with COVID-19 in the community and able to socialise face-to-face, preferences and comfort regarding online working may be different. Under other circumstances, the conveniences afforded by online working may outweigh the disadvantages. If this study were to be repeated now, more than one year after the first UK lockdown, perhaps responding would be different. Furthermore, the transition to online working was enforced, rather than a free choice, making the comparison inherently skewed. Nevertheless, issues pertaining to therapeutic relationship, safety, privacy and inequality of access of health care are likely to remain pertinent.

The current study does not address variation in clinical outcomes between face-to-face and virtual working. Pre-pandemic studies of online working using both individual and family therapies for a range of mental health problems have consistently shown comparable clinical outcomes in spite of the fact that some studies suggest that therapeutic alliance may not be as strong when working online [34, 35]. However, the extent to which this applies to the treatment of eating disorders is less clear as the studies were preliminary and also did not address issues pertaining to broader factors associated with eating disorder symptom development or maintenance, such as bio-temperamental, emotional and relational factors. These are increasingly being recognised as key areas to target in eating disorder treatment in order to prevent relapse and chronicity [36–39].

## Conclusions

Advocates for online therapy have called for the rapid transfer of mental health care online as a result of the pandemic, with some hoping it will be a catalyst for broader change in mental health service provision [27]. Clinician concern about impact on therapeutic alliance has been recognised as a barrier to the wide-spread implementation of online therapies in other areas of mental health [21]. Conversely, it has also been suggested that this is a ‘myth’, as patients tend to provide high alliance ratings [40]. The current study has highlighted disparity between ratings and more in-depth descriptions of people’s experience. Findings indicate that it will be critical to evaluate the impact of changes in therapeutic alliance, the feasibility of a range of treatment tasks, and the impact of these on outcomes in future research. Evaluating both the long- and short-term outcomes of online eating disorder treatment alongside service user and clinician experiences outside of pandemic times is necessary. This is especially needed beyond the areas in which evidence has already been collected; namely, rural and remote populations. Focused and clear attention to equality of access and social justice issues is also needed [41].

Beyond the period of lockdown enforced by the pandemic, having therapy virtually may be appropriate for *some* young people and families, at *some* stages of treatment, *if* they chose it and with attention also being paid to the well-being of clinicians.

## Supplementary Information

**Additional file 1: Supplementary Material Table 1.** Survey questions for qualitative analysis.

**Additional file 2: Supplementary material Table 2.** Thematic analysis process.

## Data Availability

Data are available from the corresponding author on reasonable request.

## Abbreviations

CAMHS: Child and Adolescent Mental Health Service
CEDS-CYP: Community Eating Disorders Services for Children and Young People
COVID-19: coronavirus disease 2019
EP: Established patients
IQR: interquartile range
MCCAED: Maudsley Centre for Child and Adolescent Eating Disorders
Mdn.: Median
NHS: National Health Service
NP: New patient
UK: United Kingdom of Great Britain and Northern Ireland
