# Supplementary material for: Is this the ‘new normal’? A mixed method investigation of young person, parent and clinician experience of online eating disorder treatment during the COVID-19 pandemic
Source: J Eat Disord. 2021 Jun 30;9:78. doi: 10.1186/s40337-021-00429-1 (PMC8243044; doi:10.1186/s40337-021-00429-1)
Supplement: Supplementary file 1 — Additional file 1: Supplementary Material Table 1. Survey questions for qualitative analysis. [file 40337_2021_429_MOESM1_ESM.docx]

Supplementary Material - Table 1 | Survey questions for qualitative analysis

| **Therapist questions**   1. Do you think there have been any benefits or advantages to conducting therapy online? 2. Do you have any concerns about conducting therapy online? 3. Are there any technical aspects of conducting therapy online that you have learnt? 4. Is there anything that you have learnt about online therapy that you would like to include in your practice when we are physically back in the clinic? 5. Is there anything else you would like to add about your experience of conducting online therapy? 6. What changes have you noticed in your own sense of efficacy or confidence since you started to deliver therapy online? |
| --- |
| **Young person and parent questions**   1. Is there anything that you have found difficult about having therapy online? 2. Is there anything that you have found helpful about having therapy online? 3. Is there anything that your/your child’s therapist could do differently that would make online therapy work better for you? 4. Is there anything else that would make online therapy work better for you? |
| *Previously engaged young people and parents only*   1. Please describe any disadvantages or losses you experienced with online therapy. 2. Please describe any benefits or gains you experienced with online therapy. |
