# Supplementary material for: Is this the ‘new normal’? A mixed method investigation of young person, parent and clinician experience of online eating disorder treatment during the COVID-19 pandemic
Source: J Eat Disord. 2021 Jun 30;9:78. doi: 10.1186/s40337-021-00429-1 (PMC8243044; doi:10.1186/s40337-021-00429-1)
Supplement: Supplementary file 2 — Additional file 2: Supplementary material Table 2. Thematic analysis process. [file 40337_2021_429_MOESM2_ESM.docx]

Supplementary material - Table 2 | Thematic analysis process

| Analysis followed the process outlined by Braun and Clarke (2006) in which the process is reflexive and includes an active role for the researcher in data interpretation and theme generation. Online surveys have been recently described as a appropriate means by which to collect rich data for this purpose. The 15-point check list for good thematic analysis process was followed. This emphasizes rigour and systematic coding and researcher reflexivity. It does not require multiple independent coders or coder agreement. The researchers took a realistic approach to this data, using an inductive and descriptive approach and seeking to remain close to participants reports. | | |
| --- | --- | --- |
| ***Phase*** | ***Process*** | ***Author Involvement*** |
| 1 Data familiarisation | Data is read repeatedly, understanding is rich and complex, and initial impressions are noted. | CS and AK engaged in data familiarisation and shared initial impressions |
| 2. Coding | Data is systematically coded- focusing on semantic meaning and remaining close to participants reports | CS and AK coded the data separately, identifying topics within the data    CS AK and JB reviewed these codes and discussed them, including identification of initial impressions of themes |
| 3. Theme Generation | Initial themes are generated from codes and coded data, with similar and related topics being clustered | CS generated initial themes |
| 4. Theme review and refinement | Initial themes are reviewed in relation to topics and dataset, themes are edited and reviewed until they fit the data | CS reviewed the coding and initial themes, and generated 3 themes that appeared to best fit the data.    These were reviewed by JB and AK against the coded data and entire dataset.    Following discussion an additional theme was generated, resulting in four final themes. |
| 5 Defining and naming themes | Each theme is refined and developed through naming and writing of the theme definition. | CS and JB named themes and wrote descriptions. |
| 6 Producing the report | Report writing provides further opportunities for refinement | CS and JB selected the illustrative quotes, and all authors reviewed and agreed. All authors were involved in manuscript writing which was led by CS. |
